# Correction of carotid artery stenosis by stent placement ameliorated paroxysmal hypertension after radiation treatment of hypopharyngeal carcinoma: a case report

**DOI:** 10.1186/s13256-022-03293-y

**Published:** 2022-02-17

**Authors:** Itzhak Brook, Ezra Cohen, Andrew Stemer

**Affiliations:** 1 Departments of Pediatrics, Georgetown University School of Medicine, Washington DC, 4431 Albemarle St NW, Washington DC, 20016 USA; 2 The Neurology Center, Washington DC, USA; 3 Departments of Neurology, Georgetown University School of Medicine, Washington DC, USA

**Keywords:** Paroxysmal hypertension, Carotid artery, Stent, Radiation, Postprandial hypotension, Hypopharyngeal carcinoma

## Abstract

**Background:**

Paroxysmal hypertension can be associated with failure of the carotid artery baroreceptors due to past exposure to radiation treatment. This report describes a patient whose repeated paroxysmal hypertensive episodes were ameliorated following placement of a carotid artery stent for the treatment of carotid artery stenosis.

**Case report:**

A 79-year-old caucasian male was diagnosed with hypopharyngeal squamous cell carcinoma (T1, L0, M0) in 2006, and received 70 Gy intensity-modulated radiotherapy in 2006 and underwent a total laryngectomy in 2008. He experienced paroxysmal hypertensive episodes since 2010 that exacerbated in frequency in 2019. Eighty percent left internal carotid artery stenosis was demonstrated by ultrasound and arteriography. Angioplasty and stenting of the left carotid artery was performed. A Doppler ultrasound study performed 5 months after the stent placement did not reveal any hemodynamic stenosis in the left carotid artery. The patient experienced postprandial hypotension and had experienced only three episodes of paroxysmal hypertension in the following 24 months. He was able to abort paroxysmal hypertensive episodes by eating warm food.

**Discussion:**

This is the first report of a patient whose paroxysmal hypertensive episodes that occurred following radiation of the neck subsided after placement of a stent in a stenotic carotid artery. The exact mechanism leading to this phenomena is unknown but may be due to several factors. The reversal of the carotid artery stent and improvement in blood flow to the carotid artery baroceptors may play a role in this phenomenon.

**Conclusion:**

The ability to ameliorate paroxysmal hypertensive episodes in a patient with carotid artery stenosis by stent placement may be a promising therapeutic intervention for paroxysmal hypertension.

## Introduction

Paroxysmal hypertension (PH, or pseudopheochromocytoma) is characterized by a sudden, temporary increase in blood pressure (BP) occurring in patients who do not suffer from pheochromocytoma [1]. PH events can last from 10–20 minutes to several hours, with varying frequency. Between these episodes, BP is usually within normal range or low, however, it may be elevated in individuals with underlying hypertension.

Neck irradiation or surgery can lead to baroreceptor failure, which can lead to PH [2]. Radiation therapy to the neck has been associated with carotid artery (CA) stenosis, creating a significant threat for individuals with head and neck cancer, including laryngectomees. The decrease in blood supply to the brain caused by CA stenosis can also generate PH episodes.

This report describes a patient whose repeated paroxysmal hypertensive episodes were ameliorated following placement of a CA stent for the treatment of CA stenosis.

## Case report

A 79-year-old Caucasian  male diagnosed with hypopharyngeal squamous cell carcinoma (T1, L0, M0) in 2006 was treated with 70 Gy intensity-modulated radiotherapy (IMRT). The cancer recurred (T2, L0, M0) 2 years later (2008) requiring bilateral radical neck dissection and total laryngectomy. Restoration of his larynx was performed using a forearm free flap. He had no signs of tumor recurrence since then. He was speaking using an esophageal–tracheal voice prosthesis.

The patient suffered from diverticulosis and migraines, and did not smoke or consume alcohol.

He developed PH 4 years after receiving the radiation therapy (2010). The episodes of PH were characterized by sudden appearance of high BP. His BP would rise from a baseline of 135/75 mmHg (pulse 80/minute) to 170/100–250/115 mmHg (pulse 95–115/minute). Most of these episodes were unprovoked, and some occurred after strenuous speech, ultrasound Doppler studies [3], or migraine headaches. The episodes occurred every 2–7 days and would last for several hours unless treated with clonidine 0.1 mg. Following initiation of the maintenance treatment with beta-blockers [atenolol 6.25 mg/daily (qd)], alpha-blockers (doxazosin 0.5 mg/qd), lisinopril (10 mg/day), and 12.5 mg hydrochlorothiazide. In 2011, the frequency of PH episodes subsided to 4–8/year. He was also treated with biofeedback therapy and counseling.

The patient also experienced orthostatic hypotension episodes. He had a positive tilt table test, and his BP dropped (> 20 mmHg systolic and > 10 mmHg diastolic within 2–5 minutes of standing.

The patient experienced dizziness, headaches, and migraines during some of these episodes. Other causes for these episodes (i.e., pheochromocytoma, renal artery stenosis, hyperthyroidism) were excluded. He did not suffer from any other cardiovascular problems.

The patients had CA ultrasound performed every 2 years since 2009. CA stenosis was found for the first time in June 2018, where the Doppler ultrasound study illustrated a 50–69% stenosis of the left proximal CA, and no stenosis greater than 50% of the right internal CA.

The patient was seen at the neurology clinic because of increased frequency of PH episodes to one every 1–2 days in the preceding 6 weeks. Physical and neurological examinations were within normal limits. He was well hydrated, BP was 145/80 mmHg, pulse was 60/minute, respiration 16/minute, and SpO_2_ 97%. An ultrasound Doppler study illustrated a critical stenosis > 70% of the left internal CA and 50–69% stenosis of the right proximal CA.

Cerebral angiogram revealed that the proximal left internal CA was 80% narrowed via NASCET criteria (Fig. 1) The proximal right internal CA had less than 50% stenosis. The left internal CA stenosis was flow limiting as most of the left hemisphere was supplied by the right internal CA via the anterior communicating artery.

**Fig. 1:**
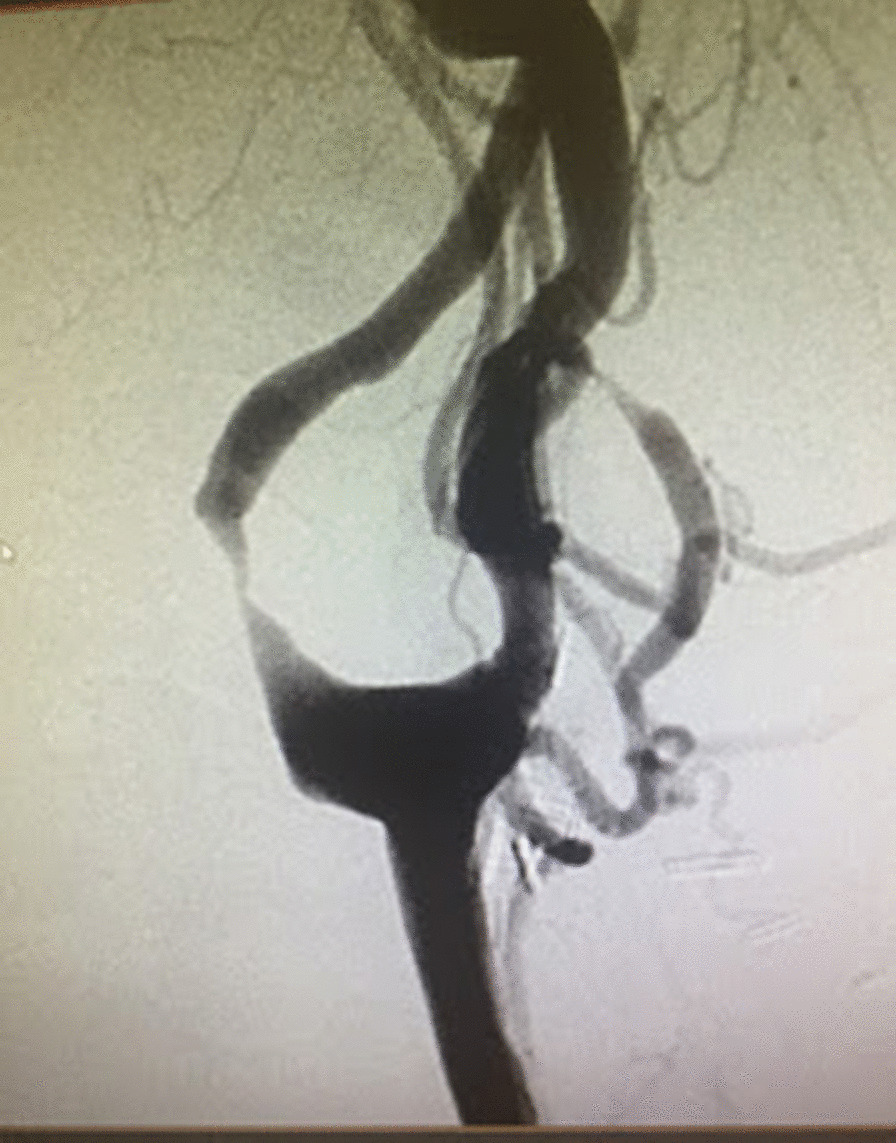
Angiogram of the left carotid artery before stenting showing 80% stenosis

The patient was placed on aspirin 320 mg and 75 mg clopidogrel/day, and 5 days later underwent general anesthesia angioplasty and stenting of the left CA. Placement of stent was done using distal protection device. A 4.0 × 30 mm balloon was used to predilate the artery before an 8–6 × 40 mm tapered XACT carotid stent was deployed. Control imaging shows no residual stenosis. Cone-beam computed tomography (CT) angiography (Dyna CT) of the brain performed following the procedure confirmed absence of infarct or hemorrhage.

CT angiography of the head and neck including maximum intensity projection (MIP) was performed 21 days after the stent placement revealed 40% stenosis of the proximal right internal carotid artery, (Fig. 2) The caliber of the left internal CA within the stent was difficult to assess. There was slight narrowing of the left internal CA as it exits the stent.

**Fig. 2:**
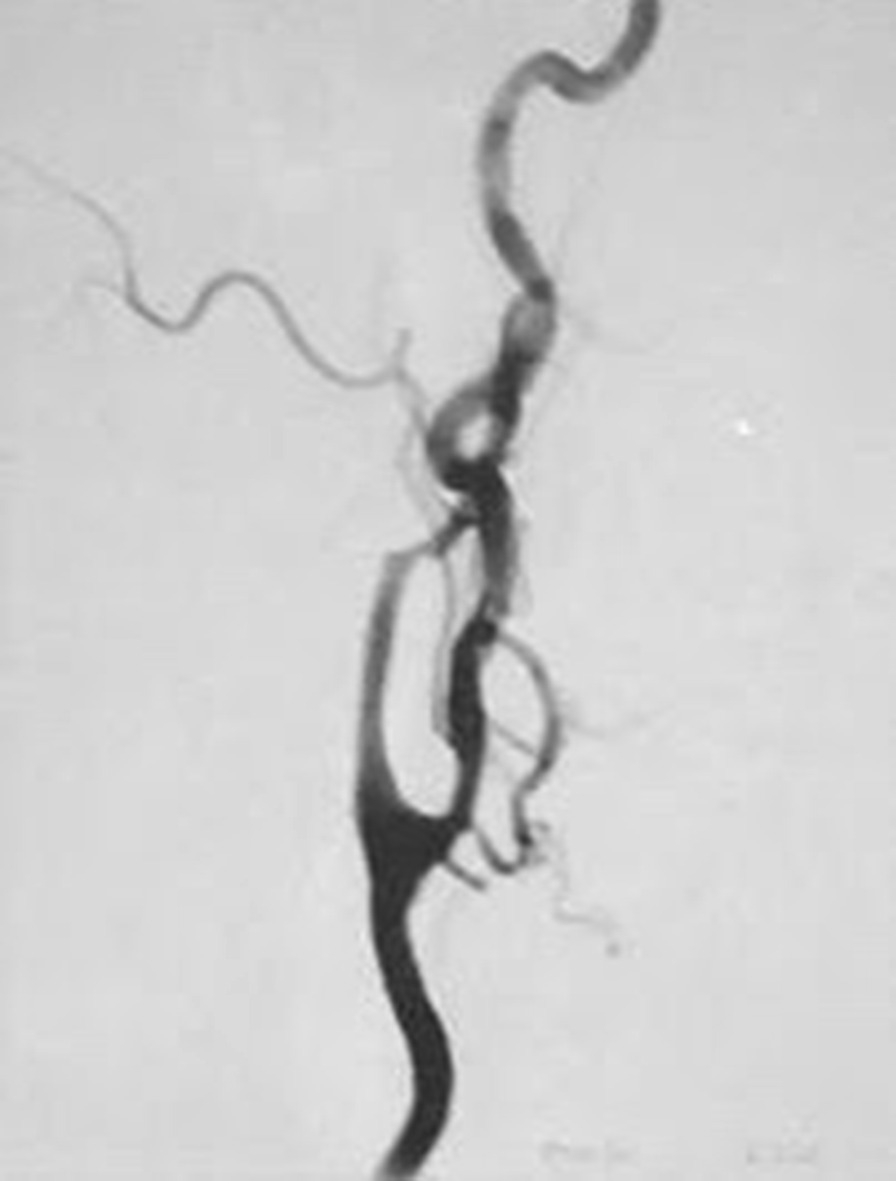
Angiogram of the left carotid artery after stenting, no stenosis

An ultrasound Doppler study performed 5 months after the stent placement illustrated mild intimal thickening and plaque formation in the right carotid bulb, without any hemodynamic stenosis. The left internal carotid artery (ICA) stent was patent with no stenosis. Vertebral flow was antegrade bilaterally.

The patient continued to take oral aspirin 320 mg and 75 mg clopidogrel/day for 6 months. Thereafter, he took only 81 mg/day.

During the month following the stent placement, the patient’s BP dropped to 110/65–117/75 mmHg and continued to range from 115/65 mmHg to 135/80 mmHg thereafter. He stopped taking hydrochlorothiazide and continued to take atenolol (6.25 mg/qd), doxazosin (0.5 mg/qd), and lisinopril (10 mg/day). The patient experienced three episodes of PH in the 24 months following the stent placement that subsided after taking oral clonidine (0.1 mg). One of the episodes occurred while the patient had a migraine episode, the second one after undergoing a urological procedure under generalized anesthesia, and the third one after receiving an injection of corticosteroids to his right shoulder joint.

The patient developed daily postprandial hypotension 18 months after the stent placement, especially after eating breakfast where his BP would drop to 92/55–110/75 mmHg. This was especially pronounced after eating warm oatmeal. The hypotension would gradually subside within 90–120 minutes. He had experienced three episodes of hypertension of 185/110–195/105 mmHg, 19 months after the placement of the stent, which he was able to abort within 10–15 minutes by eating a bowl of warm oatmeal.

We plan to follow the patient to evaluate whether the amelioration of PH will be sustained over time.

## Discussion

This is the first report of a patient whose PH episodes that occurred following radiation of the neck subsided after placement of a stent in a stenotic CA. Literature search using case report keywords as MESH words did not reveal any previous reports. The varying frequency of PH preoperatively makes the impact of stenting difficult to interpret. However, the patient had experienced PH episodes every 1–2 days prior to the placement of the sent and had only three such episodes in the 24 months after placement of the stent.

The exact mechanism leading to this phenomenon is unknown but may be due to several factors. The reversal of the CA stenting and improvement in blood flow to the CA baroreceptors may play a role in this phenomenon. The fact that PH episodes emerged in the patient 8 years before significant CA stenosis developed suggests that other factors may also exist in this patient.

Our patient experienced a decrease in his BP after the placement of CA stent. Several studies have illustrated that CA stenting leads to a reduction of BP [4–6]. Early postprocedural decreases in BP have been commonly observed after patients undergo CA stenting, and fewer patients were required to use antihypertensive agents post-CAS, compared with post-CA endarterectomy [7]. Several studies have suggested that mechanical stretch and increased distension of the carotid sinus from the compression of the stent dilatation acts is the key reason for BP decrease after CA stenting [8, 9].

Changes in baroreceptor sensitivity has also been suggested as a cause of lower BP following CA stenosis. Wustman *et al*. compared the short-term (8 and 24 hours postprocedure) effects on baroreceptor sensitivity in patients who underwent carotid endarterectomy and CA stenting procedures. The investigators found parasympathetic predominance with hypotensive effect only in patients who underwent CA stenting [10].

CA stenting plus medical treatment in patients with severe CA provided better long-term BP control compared with other medical treatments alone [11]. Chang *et al*. studied 154 patient with CA stenosis: 72 underwent CA stenting and 82 received only medical treatment [11]. Those who had CA stenting had greater BP reductions at 6 and 12 months of follow-up after adjusting for confounding factors (13.56 mmHg at 6 months, *P* = 0.0002; 16.98 mmHg at 12 months, *P* < 0.0001). This study also showed significant positive correlations between the mean or maximal systolic BP reductions 6 hours post-CA stenting, and systolic BP reductions 1 year post-CA stenting (*β* = 0.20 ± 0.07, *P* = 0.0067 and *β* = 0.47 ± 0.10, *P* < 0.0001, respectively).

Radiation to the neck has been linked to CA stenosis, and rarely to CA rupture, representing a significant risk for head and neck cancer patients, including laryngectomees [12]. Screening ultrasound within the first year since completion of radiotherapy, followed by repeat ultrasounds every 2–3 years and whenever CA stenosis  is suspected can lead to early diagnosis. Caution should be employed with Doppler ultrasound as PH may be caused by direct massage of the CA in patients who received radiation therapy for head and neck cancer [3]. The incidence of CA stenosis ranges from 18% to 38% in patients who underwent radiation treatment for head and neck cancer compared with 0–9.2% among nonirradiated patients [13]. CA disease can cause strokes and transient ischemic attack, though it does not always cause symptoms. It is important to diagnose CA stenosis or impending rupture early, before a stroke or severe bleeding has occurred.

Postprandial hypotension is common in older individuals, diabetics, and those with autonomic failure [14]. Affected individuals experience insufficient sympathetic compensation for meal-generated pooling of blood in the splanchnic circulation, inducing impaired cardiac output and systemic vascular resistance [15]. The patient was able to take advantage of his postprandial hypotension and reduce his hypertensive episodes by consuming warm food.

## Conclusions

The ability to prevent PH episodes in a patient with CA stenosis by placing a CA stent may be a promising therapeutic intervention for PH in patients with CA stenosis. Prospective studies that would evaluate the utility of this intervention are warranted.

## Data Availability

Data is available upon request

## References

[1] Zar T, Peixoto AJ. Paroxysmal hypertension due to baroreflex failure. Kidney Int. 2008;74:126–31. 10.1038/ki.2008.30 18322544

[2] Mann SJ. Severe paroxysmal hypertension (pseudopheochromocytoma). Curr Hypertens Rep. 2008;10:12–8. 10.1007/s11906-008-0005-2 18367021

[3] Brook I. Paroxismal hypertensive episodes caused by direct massage of the carotid artery by a Doppler ultrasound of the neck in a laryngectomee. J Med Ultrasound. 2020;28:114–7. 10.4103/JMU.JMU_77_19 PMC7446695 32874871

[4] Park B, Shapiro D, Dahn M, *et al*. Carotid artery angioplasty with stenting and postprocedure hypotension. Am J Surg. 2005;190:691–5. 10.1016/j.amjsurg.2005.07.004 16226941

[5] Nano G, Dalainas I, Bianchi P, *et al*. Ballooning-induced bradycardia during carotid stenting in primary stenosis and restenosis. Neuroradiology. 2006;48(8):533–6. 10.1007/s00234-006-0096-x 16670918

[6] Bussiere M, Lownie SP, Lee D, *et al*. Hemodynamic instability during carotid artery stenting: the relative contribution of stent deployment versus balloon dilation. J Neurosurg. 2009;110(5):905–12. 10.3171/2008.9.JNS08509 19231933

[7] Altinbas A, Algra A, Brown MM, *et al*. Effects of carotid endarterectomy or stenting on blood pressure in the International Carotid Stenting Study (ICSS). Stroke. 2011;42:3491–6. 10.1161/STROKEAHA.111.624478 21998053

[8] McKevitt FM, Sivaguru A, Venables GS, *et al*. Effect of treatment of carotid artery stenosis on blood pressure: a comparison of hemodynamic disturbances after carotid endarterectomy and endovascular treatment. Stroke. 2003;34:2576–81. 10.1161/01.STR.0000097490.88015.3A 14593127

[9] Mendelsohn FO, Weissman NJ, Lederman RJ, *et al*. Acute hemodynamic changes during carotid artery stenting. Am J Cardiol. 1998;82:1077–81. 10.1016/s0002-9149(98)00562-1 9817485

[10] Wustmann K, Kucera JP, Scheffers I, *et al*. Effects of chronic baroreceptor stimulation on the autonomic cardiovascular regulation in patients with drug-resistant arterial hypertension. Hypertension. 2009;54:530–6. 10.1161/HYPERTENSIONAHA.109.134023 19620513

[11] Chang A, Hung HF, Hsieh FI, *et al*. Beneficial effects of prolonged blood pressure control after carotid artery stenting. Clin Interv Aging. 2017;12:103–9. 10.2147/CIA.S122352 PMC5230727 28123290

[12] Texakalidis P, Giannopoulos S, Tsouknidas I, *et al*. Prevalence of carotid stenosis following radiotherapy for head and neck cancer: aAsystematic review and meta-analysis. Head Neck. 2020;42:1077–88. 10.1002/hed.26102 32048781

[13] Fernández-Alvarez V, López F, Suárez C, *et al*. Radiation-induced carotid artery lesions. Strahlenther Onkol. 2018;19:699–710. 10.1007/s00066-018-1304-4 29679099

[14] Pavelić A, Krbot Skorić M, Crnošija L, Habek M. Postprandial hypotension in neurological disorders: systematic review and meta-analysis. Clin Auton Res. 2017;27:263–71. 10.1007/s10286-017-0440-8 28647892

[15] Trahair LG, Horowitz M, Jones KL. Postprandial hypotension: a systematic review. J Am Med Dir Assoc. 2014;15:394–409. 10.1016/j.jamda.2014.01.011 24630686

